# Developing a Framework to Generate Evidence of Health Outcomes From Social Media Use in Chronic Disease Management

**DOI:** 10.2196/med20.2717

**Published:** 2013-08-08

**Authors:** Mark Merolli, Kathleen Gray, Fernando Martin-Sanchez

**Affiliations:** ^1^Health and Biomedical Informatics CentreThe University of MelbourneMelbourneAustralia

**Keywords:** chronic disease, social media, Internet, evidence-based practice, affordances, patient-reported outcomes

## Abstract

**Background:**

While there is an abundance of evidence-based practice (EBP) recommendations guiding management of various chronic diseases, evidence suggesting best practice for using social media to improve health outcomes is inadequate. The variety of social media platforms, multiple potential uses, inconsistent definitions, and paucity of rigorous studies, make it difficult to measure health outcomes reliably in chronic disease management. Most published investigations report on an earlier generation of online tools, which are not as user-centered, participatory, engaging, or collaborative, and thus may work differently for health self-management.

**Objective:**

The challenge to establish a sound evidence base for social media use in chronic disease starts with the need to define criteria and methods to generate and evaluate evidence. The authors’ key objective is to develop a framework for research and practice that addresses this challenge.

**Methods:**

This paper forms part of a larger research project that presents a conceptual framework of how evidence of health outcomes can be generated from social media use, allowing social media to be utilized in chronic disease management more effectively. Using mixed methods incorporating a qualitative literature review, a survey and a pilot intervention, the research closely examines the therapeutic affordances of social media, people with chronic pain (PWCP) as a subset of chronic disease management, valid outcome measurement of patient-reported (health) outcomes (PRO), the individual needs of people living with chronic disease, and finally translation of the combined results to improve evidence-based decision making about social media use in this context.

**Results:**

Extensive review highlights various affordances of social media that may prove valuable to understanding social media’s effect on individual health outcomes. However, without standardized PRO instruments, we are unable to definitively investigate these effects. The proposed framework that we offer outlines how therapeutic affordances of social media coupled with valid and reliable PRO measurement may be used to generate evidence of improvements in health outcomes, as well as guide evidence-based decision making in the future about social media use as part of chronic disease self-management.

**Conclusions:**

The results will (1) inform a framework for conducting research into health outcomes from social media use in chronic disease, as well as support translating the findings into evidence of improved health outcomes, and (2) inform a set of recommendations for evidence-based decision making about social media use as part of chronic disease self-management. These outcomes will fill a gap in the knowledge and resources available to individuals managing a chronic disease, their clinicians and other researchers in chronic disease and the field of medicine 2.0.

##  Introduction

### Overview

Presently, no rigorous frameworks exist informing how to generate evidence of improved health outcomes from social media use in chronic disease management based on robust research design. We wish to address this problem. The work presented in this paper is part of a larger research project, which has two aims. First, on a theoretical level it aims to develop a framework for generating evidence of improved health outcomes from social media use as part of chronic disease self-management. Second, on a practical level it aims to produce a series of recommendations for clinicians suggesting evidence-based decision making about social media use in the same setting (ie, best practice for using social media). We also recognize the shortcomings of much of the research in this domain. Namely, there is a failure to discuss and unpack fundamentals within the research context. In the case of this research, addressing the above study aims requires careful consideration of the following key concepts: social media, evidence-based practice (EBP), affordances, and patient-reported outcomes (PRO). They will be outlined and described in more detail.

### Background

#### Social Media in Chronic Disease Management

Attempts to ratify definitions of social media remain problematic, partly because agreements remain elusive. However, the underlying principles of communication, participation, collaboration and user-centeredness are commonalities [1]. Social media are essentially the services that foster the aforementioned activities and examples of platforms include: social network sites (SNS), blogs, wikis, and video sharing services to name a few [2,3]. In their current form, they may be seen as more highly evolved relatives of Internet 1.0 applications, displaying high social functionality and interaction [4]. 1.0 Internet applications can be seen in simple email and basic websites designed essentially for sourcing information, not creation and sharing [4].

People dealing with chronic disease are increasingly communicating their health concerns online, with poorer health status, stigmatization, isolation, and disconnection outlined as major reasons [5]. Social media have created new opportunities for management, not only for the way in which patients self-manage their conditions but also for clinicians who treat them [5]. These platforms allow patients to choose how they share and receive health information, creating a greater user-centric, engaged, and collaborative experience [6].

#### Evidence-Based Practice

Despite the apparent infiltration of the social Web into chronic disease management, there is a challenge for clinicians who wish to use social media in patient management within an evidence-based practice (EBP) framework. The challenge is establishing robust recommendations for their use based on best available evidence, while also taking into consideration both clinician expertise and patient preferences [7,8]. We therefore pose the question: How can social media use in chronic disease be approached in a more evidence-based manner?

EBP refers to providing the most effective care to patients based on the best available evidence [7,8]. While this seems self-explanatory, in practice implementing EBP is not always so simple. Traditionally EBP requires clinicians to have the necessary skills, time and effort to sort through research and implement it with their patients. These factors are one possible reason that EBP is not always adhered to clinically [7].

A four-step process [7] suggests that to improve clinician adherence to EBP, the clinician: asks an answerable question about the problem, finds best evidence for management, critically appraises the evidence and integrates it with the unique needs of the patient in mind. However, in order to do this and satisfy EBP principles, a further set of steps [7] outlines that clinicians need to: be aware of valid evidence, accept the evidence to change practice preferences, correctly apply the evidence, have the necessary tools and resources available to do so, act upon the evidence, inform and agree upon treatment with the patient and have patients adhere to the course of action.

This conventional approach to EBP provides the scope for the current research and is applicable to how social media in clinical practice may be considered. As highlighted above, evidence-based decision making about social media use in chronic disease to improve health outcomes also relies not only on an efficient set of processes but on the best available information and guidance being available to clinicians, researchers and patients to make informed decisions [8]. However, the current problem faced with social media use is the relative paucity of high quality literature definitively examining its use in chronic disease management, specifically regarding their effectiveness to improve health outcomes and, therefore lack of research to reliably inform these decisions [9]. One might argue that our knowledge of social media use in chronic disease does not support EBP and needs further refinement.

Offline approaches to chronic disease management have been commonly “unidirectional”. This means they have emphasized clinical research findings above all else, suggesting research should inform clinical practice [8]. However, advancements in technology have caused such processes to evolve. Information flows are now more circular and incorporate information from a variety of sources to inform EBP. Such sources of information include clinician expertise, clinician experience, patient views and patient preferences as part of the decision-making process [8]. Social media use may be broached in the same way. The advent of the social Web represents a shift in how evidence of health outcomes in chronic disease can be generated, as patients are choosing how and when they access information to help manage their condition. It presents a culture of “shared responsibility” among multiple stakeholders [10].

#### Therapeutic Affordances

“Affordance” as a theory may be relatively foreign to health care and more specifically to this research in understanding how social media are used in chronic disease management to affect health outcomes. Notably, this is perhaps because its origins are from perceptual psychology. However, it has been appropriated to human-computer interaction [11,12]. The idea underlying affordance is that it attempts to explain how people perceive things in their immediate environment differently, perceiving what an object is potentially useful for, not simply what it is [11]. It is for this reason that people must first perceive what an object can be used for before they interact with it. Affordances are perceived uniquely by each of us, suggesting why some people use the same objects differently to others [11]. Within a technology setting, the affordance concept is further refined. While the idea behind an object’s actionable possibilities needing to be perceived is important, the emphasis is placed on the unique relationship that exists between the object and the individual [12]. Greater emphasis is therefore placed on past experiences, end goals for use and one’s belief/value system. Essentially the individual’s goal and context for use will lead to a different perception of the affordances [12,13].

Contemplating the importance of the therapeutic affordances of social media has been a key motivation behind our work. We theorize that different social media interactions can precipitate different effects for different people self-managing chronic disease. We anticipate that this approach may help to guide researchers when conducting research projects in this domain and also guide clinicians when deciding whether social media may form a meaningful part of patient management.

#### Patient-Reported Outcome Measurement

Patient-reported outcome (PRO) measurement has long been an accepted means to evaluate the success of medical interventions and present evidence of changes to health outcomes. This approach is intended to foster the patient’s perspective of an intervention via outcome measure questionnaires [14]. They provide quantitative data from a patient’s responses to allow the researcher to measure change from the patient’s own perspective, essentially providing a means to quantify qualitative information [14].

The chronic disease landscape in particular pushes us to establish valid PRO measurement research methodologies. The breadth of chronic conditions (eg, chronic pain, cancer, diabetes, arthritis, depression, fibromyalgia, etc) creates a relative lack of consistency in regards to the measures chosen to assess health outcomes. In chronic disease, PRO tools are generally designed to assess functional limitations, symptoms, health status and health-related quality of life (HRQL) [15]. Common questionnaires that have been utilized in studies of social media include: the visual-analogue pain rating scale, profile of mood states, depression anxiety and stress scales and the SF-36 [16]. This variety highlights the need for validated PRO tools to address this problem, allowing research findings to be standardized, generalized and comparable across a range of chronic diseases and different studies.

## Methods

To consider how to generate evidence of health outcomes from social media use we propose a dual method that harnesses both qualitative and quantitative research findings and allows them to be combined.

The first part of the method focuses on *identification and examination of the therapeutic affordances of social media* that can help to explain how use of these platforms may underlie favorable health outcomes.

We feel that it is important to examine more closely by what mechanisms social media actually impact health outcomes. As implied in [9], research to date has not adequately examined patient perceptions towards different media and their effect on health outcomes. Propositions are made that future social media research in this domain should consider frameworks that may be used to approach and evaluate what components of social media interventions are best suited to different patient contexts and needs. This approach may help bolster a more effective combination of both online and offline support in chronic disease self-management [9]. It is here that we believe examination of the therapeutic affordances of social media may hold valuable information.

We have conducted an extensive review that has been published, of empirical and theoretical literature in order to define potentially therapeutic affordances of social media in chronic disease management [17]. The findings of this review formed the foundations for an online survey we have recently closed that targeted approximately 200-250 people with chronic pain (PWCP), recruited from large online health networks, smaller online pain support communities and chronic disease organizations, as well as international pain organizations. The survey and its findings will be an important next step in development of this framework, as it aims to refine our findings and understanding of individual perceptions towards health outcomes experienced from use of social media (specifically considering these therapeutic affordances).

Chronic pain has been selected as a suitable subset of chronic disease self-management for our study purposes. The reason is because while chronic pain is a recognized chronic disease in its own right, it is also a common manifestation or comorbidity of many other chronic diseases. This provides us an opportunity to generalize across various chronic diseases in the clinical setting. This is further highlighted in the same literature review we have conducted, presenting examples of various social Web-based interventions impacting health outcomes in chronic pain related studies [17].

The other part of the method focuses on *validated and appropriate outcome measurement* to reliably assess health outcomes from social media use (that more specifically considers these affordances).

Pertinent to our current research is that social media’s validity as chronic disease management tools is uncertain and still largely untested. Formal measurement of health outcomes is required to actively assess whether social media interventions are effective for improving health outcomes in chronic disease [15]. In our case, we refer to tailored interventions specifically taking into consideration the therapeutic affordances of social media. In order to measure effectiveness rigorously, both qualitative and empirical information about these affordances, combined with validated PRO measurement, are required to assess effect on health outcomes. We require an instrument that has been shown to be valid and reliable to assess PRO across a range of chronic diseases and for a range of different outcomes. We plan to test the ability of one such tool (to be further described in this paper) to produce clinically significant and replicable evidence of health outcomes from social media studies considering therapeutic affordances.

## Results

### Evidence of the Therapeutic Affordances of Social Media in Chronic Disease Management

Our literature review identified evidence of self-reported health outcomes and other effects seen from social media use in different chronic disease scenarios. This evidence is presented in full in the review, which has been published elsewhere [17]. To briefly summarize, we were able to highlight associations between various social platforms and improved health outcomes. However, relationships and linkages are more difficult to infer. Without closer evaluation, review tenuously explained the connection between platforms and outcomes, doing little to describe what patients attribute any improvements to or how social media meet their individual needs. Upon closer investigation it was possible to qualitatively identify a series of therapeutic affordances that we hypothesize may better explain mechanisms behind how social media have an effect on health outcomes. The affordances that appear significant in this regard we have labeled: identity, flexibility, structure, narration and adaptation [17]. These therapeutic affordances form the core information we are further exploring in the online survey. We will refine them and further examine their presence or absence via the aforementioned survey results to enable us to explore their perceived value in more detail before formal clinical effectiveness can add further validation via a planned pilot intervention. While we expect that different researchers and clinicians will have their own opinions and ideas regarding social media’s affordances, we believe this structure presents a robust approach for generating evidence of health outcomes from social media use.

### Measuring the Effectiveness of the Therapeutic Affordances of Social Media: PRO Measurement

We have decided to explore and utilize a particular instrument of PRO measurement, the Patient-Reported Outcomes Measurement Information System (PROMIS). We are doing this because PROMIS is an item bank system of commonly studied PROs that has been tested and calibrated, demonstrating good reliability and validity across a range of chronic diseases, and shows moderate to strong correlations with other common outcome measures [18]. PROMIS provides great scope for this research as its generalizability has the advantage of allowing comparability across a range of chronic diseases, as item banks are not designed to differentiate subtypes of symptoms from different diseases (ie, pain in fibromyalgia vs pain in arthritis for example) [18]. Rather, they aim to delineate based on severity of symptoms or impairment of function. The focus is on physical, mental, and social health (including sub domains of: physical function, pain, distress, fatigue, social function, global health, etc). The aim is that this would be appropriate for patients with a wide range of chronic diseases [18] and has the potential to address the generalizability and consistency issues that come from combining two complex areas—chronic disease and social media.

##  Discussion

### Overview

While literature exists outlining health outcomes from social media use, few attempts have been made to investigate how social media operate to meet the specific and individual needs of different chronic disease patients. As more social media uses emerge and further reports are published, researchers will require even more comprehensive methodologies and meta-analytic research designs to synthesize collective knowledge in the quest towards incorporating social media use into EBP [9,10]. The information presented in the results section forms the basis of our proposed framework below.

### Our Proposed Framework

**Figure 1:**
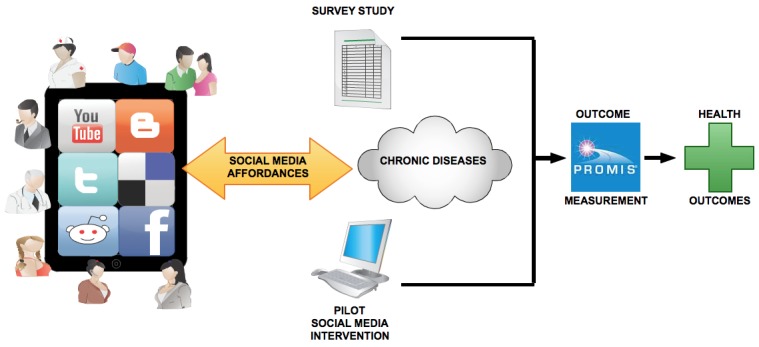
Generating evidence of health outcomes from social media use.

Our proposed framework represents a research approach for generating evidence of health outcomes from social media use in chronic disease management (Figure 1). Its design also provides the basis for evidence we expect to see of health outcomes from social media use; as well it forms the basis for informing practical recommendations for health professionals to assist them with their decision-making about social media use for patient self-management.

The proposed framework follows several steps. First, taking into account the uses and interactions social media affords people with chronic disease, the framework begins with a thorough review of the literature of social media use in chronic disease management [17]. Second, people with chronic disease are *surveyed* regarding their perceptions of the therapeutic affordances of social media and how social media use may lead to health outcomes. Then, they undergo an *online pilot intervention* testing how social media can be targeted (considering these affordances) to better tailor management to individual needs. Finally, PRO from both survey and online intervention are measured using specific item banks from PROMIS to provide empirical evidence of health outcomes. Using standardized PROMIS item banks allows for health outcome questions in the survey and online intervention to be tailored depending on the chronic disease being studied, as well as the primary outcome measure of interest (ie, pain interference, physical function, mood, cognition, sleep, QOL, etc) [18].

### Progress to Date and Future Directions

As outlined, progress to date has expressed the complexities involved and how important it is to be systematic when approaching the study of health outcomes in chronic disease using social media. This is why we feel research would benefit from frameworks detailing the evidence synthesis process. We began our research by examining the chronic disease landscape to gain a deeper knowledge of management both offline and online. This enabled a more focused approach to then explore social media (culminating in the literature review that we have alluded to [17]). We emphasize and introduce the concept of “therapeutic affordances” of social media because current research lacks discussion of the connection between use and health outcomes. Despite affordances being a somewhat enigmatic construct, the affordance approach to social media has recently been studied in a similar fashion across other domains. For example, one study published in peer-reviewed literature describes affordances in organizational communication processes [13]. We bring the same concept to chronic disease management and hope that its applicability can be further explored within a variety of health scenarios.

The global online survey is now closed and recruited 231 participants. In the coming months we plan to present findings of the survey and describe them in a future paper, which is currently beyond the scope of the present paper. We anticipate the results will provide a refined understanding of both how social media affect health PROs and also how people with chronic pain perceive this to be so. The steady flow of contact and interest in the survey, and coherence of preliminary results indicates that participants have been able to follow and make sense of the work. This provides early evidence and validation supporting the theoretical basis of our framework. Unfortunately, this is not expected to be rigorous enough to inform decision-making about social media use in the clinical setting. Future plans of our research are to conduct a pilot intervention in order to further develop and validate our framework and bring us one step close to evidence-based decision making about social media use in chronic disease self management.

### Strengths and Limitations

Standardized outcome measurement lies at the heart of bringing our research together and without it the reliability, validity, and generalizability of this project will be of limited value. For this reason we have selected and discussed PROMIS as the outcome instrument we are using to investigate the PROs in both our survey and also the pilot intervention. PROMIS has many strengths that suit this research. Its item banks (or outcome domains) can be translated into “short-forms” of targeted questions to suit any study, its item banks have been tested amongst large heterogeneous patient cohorts and they have been tested against other commonly used outcomes measures [18]. However, perhaps the biggest strength of PROMIS lies in its ability to be applied to a wide range of chronic conditions and to measure a wide range of functional outcome domains, correlating strongly with all. No questions are specific to any one cohort of patients, they are generalizable and therefore permit a large range of participants to supply PRO data without needing to be too disease specific [18]. For this reason we believe it is ideal as it can fit into the survey approach and then be cross-referenced to a pilot intervention. This also allows for researchers conducting future studies into the health outcomes from social media use in chronic disease to substitute the functional outcome domain to reflect their own study’s interests and needs.

Conversely, we acknowledge that while initial PROMIS item banks have been shown to display reliability, validity and accuracy when compared to other common outcome measures, longitudinal data is still to be finalized. PROMIS researchers are confident however, that this will also be shown [18].

### Future Considerations

As research and clinical practice progresses, the challenge for clinicians who plan to use social media in patient management or recommend them as part of individual self-management will persist. This will continue unless research into social media in this domain establishes evidence-based frameworks [8]. While we have previously found that there is a paucity of rigorous studies investigating the health outcomes of social media use [17], a 2013 study investigating Web 2.0 chronic disease self-management has been published that goes some way to addressing this [9]. The authors propose use of the Reach, Efficacy, Adoption, Implementation, and Maintenance (RE-AIM) framework for “evaluating” the effectiveness of Web 2.0 interventions in a methodical evidence-based fashion. The framework is described in [19] and is a five-step method that describes the reach, efficacy, adoption, implementation, and maintenance of social media interventions for chronic disease self-management. It is hypothesized that visiting the RE-AIM framework may be helpful to develop social media interventions that are more likely to be adopted in practice [9,19,20]. It is certainly worthy of further consideration. The aim of the current research is to support the same spirit of evidence-based Web 2.0 interventions in clinical practice, thus making social media use in chronic disease management more accountable. Evidence of the benefits and/or limitations of social media use will greatly enhance the potential of these technologies in the future. However, while the RE-AIM framework is used for study “evaluation” purposes, our research puts forward a unique “evidence-generation” framework for consideration in future studies. This is to aid in informing research design from the early research phases, not just at the intervention success evaluation stage.

### Conclusions

Our research to date on framework development for conducting research into health outcomes in chronic disease centers on a deeper investigation of the therapeutic affordances of social media in this context. Second, we emphasize the importance of valid and standardized PRO measurement. Together, affordances and PRO form the basis of a novel methodological approach for how to generate evidence of health outcomes from social media use, as well as clinical recommendations for evidence-based decision-making about social media use in chronic disease management. With further work and collaboration, this research method and framework may aid research design for social media interventions and allow for greater improvements in health outcomes to be recognized.

## Abbreviations

EBP: evidence-based practice
HRQL: health-related quality of life
IBES: Institute for a Broadband Enabled Society
PRO: patient-reported outcome
PROMIS: Patient-Report Outcome Measurement Information System
PWCP: People With Chronic Pain
RE-AIM: Reach, Efficacy, Adoption, Implementation, and Maintenance
SNS: social network sites
